# Molecular Machines and Microrobots: Nanoarchitectonics Developments and On-Water Performances

**DOI:** 10.3390/mi14010025

**Published:** 2022-12-22

**Authors:** Katsuhiko Ariga

**Affiliations:** 1 International Center for Materials Nanoarchitectonics (WPI-MANA), National Institute for Materials Science (NIMS), 1-1 Namiki, Tsukuba, Ibaraki 305-0044, Japan; ARIGA.Katsuhiko@nims.go.jp; 2 Department of Advanced Materials Science, Graduate School of Frontier Sciences, The University of Tokyo, 5-1-5 Kashiwanoha, Kashiwa, Chiba 277-8561, Japan

**Keywords:** micromachine, microrobot, molecular machine, nanoarchitectonics, air-water interface

## Abstract

This review will focus on micromachines and microrobots, which are objects at the micro-level with similar machine functions, as well as nano-level objects such as molecular machines and nanomachines. The paper will initially review recent examples of molecular machines and microrobots that are not limited to interfaces, noting the diversity of their functions. Next, examples of molecular machines and micromachines/micro-robots functioning at the air-water interface will be discussed. The behaviors of molecular machines are influenced significantly by the specific characteristics of the air-water interface. By placing molecular machines at the air-water interface, the scientific horizon and depth of molecular machine research will increase dramatically. On the other hand, for microrobotics, more practical and advanced systems have been reported, such as the development of microrobots and microswimmers for environmental remediations and biomedical applications. The research currently being conducted on the surface of water may provide significant basic knowledge for future practical uses of molecular machines and microrobots.

## 1. Introduction: Nanoarchitectonics

**Figure 1:**
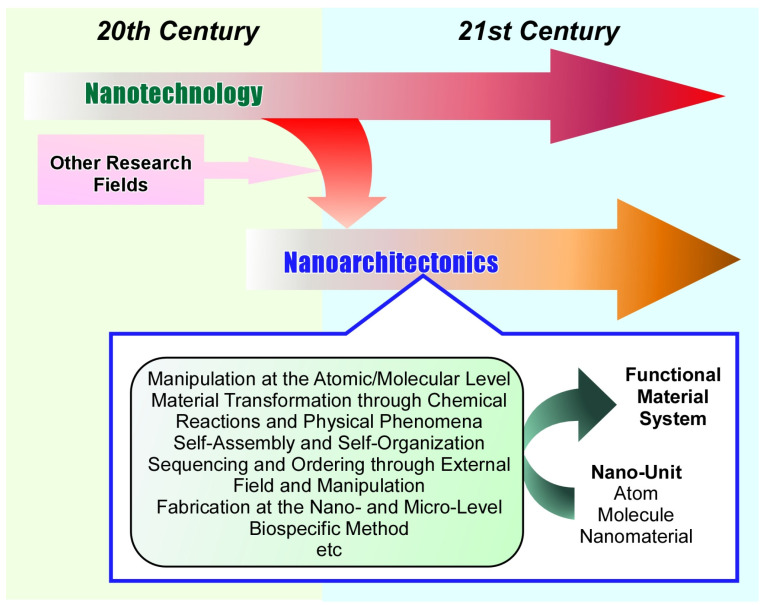
History and outline of the nanoarchitectonics concept.

The development of mankind depends on the exploration of new functional materials. In particular, it has been supported over the last century by the development of various research fields related to materials development, including organic chemistry [1,2,3,4], inorganic chemistry [5,6,7,8], polymer science [9,10,11,12], coordination chemistry [13,14,15,16], supramolecular chemistry [17,18,19,20], biochemistry [21,22,23,24], and the other materials sciences [25,26,27,28]. The later period of the 20th century saw the inception of the revolutionary concept of nanotechnology. Nanotechnology enabled direct observation [29,30,31], functional evaluation [32,33,34], and manipulation [35,36,37] at the atomic, molecular, and nano-level, and greatly advanced our understanding of science at the nano-level. Along with this, it has become clear that the functions of various materials are highly dependent on their internal nanostructures. A common understanding has emerged that it is not only the characteristics of the material itself, but also how to rationally design and control its nanostructure that is so important for the development of functional materials [38,39,40]. In other words, the nano-related knowledge that nanotechnology has elucidated should be applied to material science. The nanoarchitectonics was proposed as a methodology to architect functional material systems from nano-units (atoms, molecules, and nanomaterials) by integrating nanotechnology and various material sciences and related fields (Figure 1) [41,42]. While Richard Feynman proposed nanotechnology in the 20th century [43,44], nanoarchitectonics was proposed by Masakazu Aono at the beginning of the 21st century [45]. Nanoarchitectonics is considered to be a post-nanotechnology concept [46].

Nanoarchitectonics combines various processes from basic units such as atoms, molecules, and nanomaterials to architect functional material systems. It selects and combines methods such as manipulation at the atomic/molecular level, material transformation through chemical reactions and physical phenomena, self-assembly and self-organization, sequencing and ordering through external fields and manipulations, fabrication at the nano- and micro-levels, and biospecific methods to create functional materials [47]. Compared to materials fabrication by self-assembly, which relies on simple equilibrium processes, nanoarchitectonics is advantageous in forming asymmetric and hierarchical structures [48] because it often incorporates several processes, including equilibrium and nonequilibrium ones. For example, various unit processes such as self-assembly [49,50,51], template syntheses [52,53,54] can be combined with thin films using methods such as the Langmuir-Blodgett (LB) method [55,56,57] or layer-by-layer (LbL) assembly [58,59,60]. Through molecular self-assembly together with template synthesis, synthesized nanomaterials can be immobilized on device surfaces as hierarchical thin films using these methods [61,62]. This methodology can be applied regardless of the type of material or application. Since materials are originally made of atoms and molecules, nanoarchitectonics is a method that can be applied to virtually any types of material fabrication. It can be likened to the Theory of Everything in the world of physics [63], and can be called the Method for Everything in materials science [64]. In fact, the concept of nanoarchitectonics is used in fundamental areas such as fabrication and control of materials [65,66,67], micro/nano-structure organization [68,69,70], understanding of physical phenomena [71,72,73], and pursuit of basic biological and biochemical functions [74,75,76], as well as application-oriented fields such as catalysis [77,78,79], sensors [80,81,82], devices [83,84,85], energy-related applications [86,87,88], environment-related applications [89,90,91], and biomedical applications [92,93,94].

Thus, nanoarchitectonics shares values with existing fields of science and technology. Therefore, some of the existing research may not be usually called nanoarchitectonics, but may take a similar approach. In particular, functional materials are currently being developed based on nanostructure control, including various supramolecular assemblies, complex structure formation, and polymer alignment. In some of these examples, we can find important elements of nanoarchitectonics. Tohnai et al. have performed structural nanoarchitectonics of functional organic molecules in solids [95]. Using organic salts of 2,2’-bithiophene and disulfonic acid as functional excimer moieties and using them as sequence control moieties. The intensity of excimer emission was controlled by regulating the mobility of π-conjugated molecules through nanoarchitectonics in solids. Some have attempted to architect polymer structures through supramolecular interactions. Harada et al. used the molecular recognition function of cyclodextrins to architect supramolecular polymers and related materials [96]. Through main chain recognition, polyrotaxanes, tubular polymers, supramolecular machines, and artificial enzymes were nanoarchitected. Self-healing and muscle-like materials were also developed. Haino et al. constructed chiral supramolecular polymers through the formation of host-guest complexes of chiral diammonium guests with octaphosphonate biscavitands as hosts [97]. Atomic force microscopy revealed both cyclic oligomers and supramolecular polymer chains. Structural information is also transferred from nanoarchitectonically fabricated structures to other materials. The incorporation of other materials into the control structures created by nanoarchitectonics can also control the physical properties of those materials. As summarized in a recent review paper by Hosono and Uemura [98], the pore channels of metal-organic frameworks (MOFs) can incorporate polymers in elongated conformations that are otherwise difficult to obtain. This also makes it possible to study the properties of single polymer chains. In a recent review, Miyasaka describes the architecture and property control of MOFs incorporating electron donors and electron acceptors [99]. By changing the electronic structure of the internal electron-donor and electron-acceptor subunits, i.e., by selecting the electron-donor and electron-acceptor, the charge distribution between them was precisely tuned. Structure and function have also been controlled by external stimuli. For example, the review by Seki and co-workers discusses the control of photoresponsive polymer aggregates [100]. The self-building of surface morphology by single laser beam irradiation, phototriggered migration by Marangoni flow, surface relief grating formation, and polymer brush function are discussed, using polymers and supramolecules containing azobenzene units.

As seen in the above-mentioned examples, nanoarchitectonics and its related chemistry, such as supramolecular chemistry, have made significant achievements in functional materials systems. However, since nanoarchitectonics is based on nanotechnology, it is also powerful in the fabrication and functional development at much smaller levels. For example, in a technique known as local probe chemistry, the tip of a probe microscope can be used to carry a reactive molecule to a peep site and cause a substitution reaction [101,102]. Nanoarchitectonics at the molecular level can be performed with nanotechnology tools. This is a fusion of nanotechnology and organic chemistry and may be a typical example of nanoarchitectonics at the smallest level. In addition, in a technique known as on-surface synthesis, the chemical reaction itself can proceed while observing molecules on a solid surface [103,104]. This is also molecular nanotechnology, a fusion of nanotechnology and organic chemistry. As seen in these examples, nanoarchitectonics is also powerful in the nano realm [105]. More fundamental in such systems are those in which molecules and supramolecules themselves function like machines. As the name suggests, a molecular machine is one in which the molecule itself works like a machine, but instead of simple molecules, units are used that are built into machines, such as catenanes, rotaxanes, and molecular shuttles [106,107,108]. These are molecular level architectures and may be considered the products of molecular level nanoarchitectonics. There are many recent examples where several molecular rotors are architecting several units, such as molecular gears [109,110].

Nanoarchitectonics produces functional systems of various sizes based on nano-level phenomena. Nano-level phenomena often have effects of ambiguities such as thermal fluctuations, stochastic distributions, and quantum effects. Therefore, nanoarchitectonics can be considered to harmonize effects including ambiguities rather than the sum of their respective effects [111]. In particular, nanoscale objects, such as molecular machines, are often subject to nanoscale-specific effects, and this is an area where the concept of nanoarchitectonics can be brought into play. Advances in observation and evaluation techniques have made it possible to directly observe the behavior of molecular machines. For example, nanocars, in which molecules and their aggregates move like a car, are such targets [112,113,114]. The motion of nanocars on solid substrates is not simple and is affected by various nanoscale-specific phenomena. These observations are often made at solid interfaces and under cryogenic and high vacuum conditions. If we set this up in a more practical field, such as a biomembrane or liquid interface, the effects of the fluctuations are immeasurable. However, there remain many unsolved problems there, and it is an interesting frontier [115,116].

From the above perspective, this review paper will focus on micromachines and microrobots [117,118], which are objects at the micro-level with similar machine functions, as well as nano-level objects such as molecular machines and nanomachines [119,120]. In particular, their behavior at liquid interfaces (mainly at the air-water interface) will be discussed from a nanoarchitectonics perspective. The paper will initially review recent examples of molecular machines and microrobots that are not limited to interfaces, noting the diversity of their functions. Next, examples of molecular machines and micromachines/microrobots functioning at the air-water interface will be discussed. The air-water interface is an old medium with more than 100 years of history in the study of LB membranes [121,122]. However, it is also a frontier field for cutting-edge subjects such as nanoarchitectonics, molecular machines, nanomachines, micromachines, and microrobots. This review paper would like to discuss the new problem of how molecular machines and microrobots work at the traditional but frontier field of air-water interface.

## 2. From Recent Examples

### 2.1. Molecular Systems and Molecular Machines

The development of molecular machines and similar functional molecules is extensively researched. It is virtually impossible to present an exhaustive list of all examples. Here, a few recent examples are given to illustrate the breadth of the developments.

Molecular geometry plays an important role in the mechanical functioning of molecules. Tokunaga, Tahara, and co-workers present a theoretical consideration of the molecular geometry required for quantum dot cellular automata [123]. They showed how classical electrostatics combined with density functional theory calculations can be used to aptly characterize molecular geometries for device design. Applied to a library of biferrocenium dimers, it was found that covalently bonded parallelogram dimers respond correctly to six different patterns of nanoscale electric fields. Their correct functioning as device cells for AND and OR logic gates was also theoretically demonstrated. These attempts address the practical problem of distortion from the ideal square cell structure. In addition, these approaches can be considered as essential steps to provide logical functionality to the molecule.

**Figure 2:**
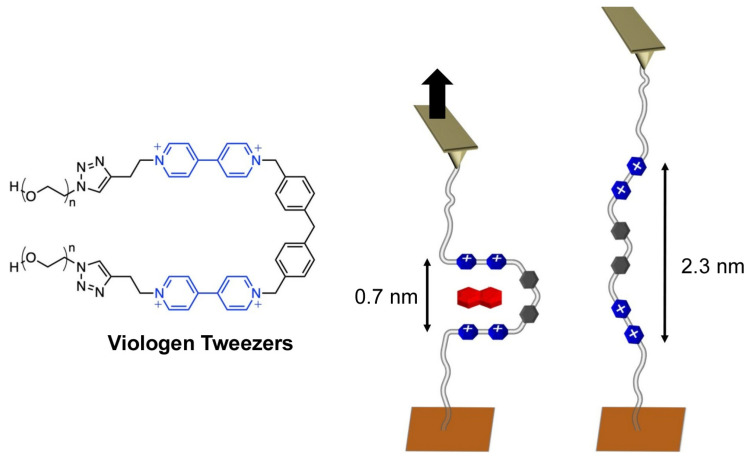
Viologen tweezers sandwiched between the AFM tip and a mica substrate for quantification of the force of various weak noncovalent interactions produced by molecular machines at the single molecule level. Reprinted with permission from Reference [124]. Copyright 2020 American Chemical Society.

Direct evaluation of the mechanical motion of molecular machines has also been done. The donor-acceptor π interaction is often used in supramolecular and molecular machine design. In addition to interpreting these interactions in terms of energies obtained at thermodynamic equilibrium, evaluation of the mechanical strength of processes out of equilibrium is also necessary for implementing synthetic molecular machines. Sluysmans et al. used single molecule force microscopy to determine the non-equilibrium mechanical strength of individual donor-acceptor π interactions [124]. As shown in Figure 2, molecular tweezers composed of PEG-tethered viologen are sandwiched between the AFM tip and a mica substrate. Measurements of this process yield force-distance properties. This technique makes it possible to quantify the force of various weak noncovalent interactions, not only donor-acceptor π interactions. It will be possible to quantitatively evaluate the details of the potential work produced by molecular machines at the single molecule level.

**Figure 3:**
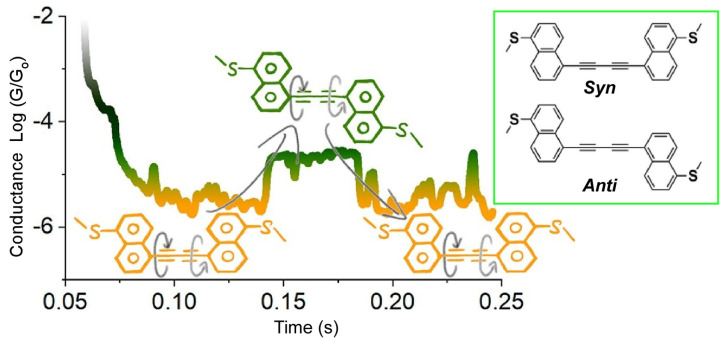
Single molecular conductance measurements for a cranked molecule, in which two naphthyl groups are free to rotate along the 1,3-butadinyl axis, to evaluate fluctuations corresponding to the syn- and anti-conformation change. Reprinted with permission from Reference [125]. Copyright 2021 American Chemical Society.

Zhang, Hong, Chen, and co-workers evaluated the internal dynamics of rotation in molecular machines at the single-molecule scale (Figure 3) [125]. They developed a cranked molecule in which two naphthyl groups are free to rotate along the 1,3-butadinyl axis, inducing a conformational inversion between syn and anti conformers. Single molecular conductance measurements were performed using scanning tunneling microscopy break junction methods and theoretical calculations. Conductance fluctuations corresponding to the syn- and anti-conformations were evaluated. Theoretical calculations suggest that the fluctuations are due to intra-orbital interference upon the energy shift of the frontier orbital associated with conformational inversion. This system verifies that the measurement of single-molecule conductance is a reasonable way to monitor the rotation of molecular machines. They also showed that molecular rotation can modulate the single-molecule conductance, providing fundamental knowledge for the design of molecular devices.

**Figure 4:**
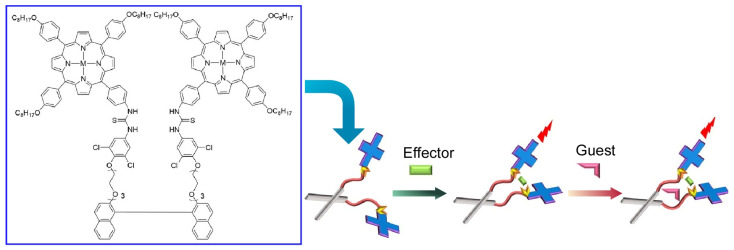
Chemosensor controlled using chiral dianions as effectors to be capable of second excited-state fluorescence upon guest binding. Reprinted with permission from Reference [130]. Copyright 2022 Chemical Society of Japan.

Molecular recognition is a key in functional systems such as molecular machines and molecular sensors [126,127,128]. Related research has been widely conducted. For example, in a recent review by Kumar, novel urea/thiourea-based receptors were developed to develop detection systems for diverse substances such as fluoride, cyanide, and tabun (first nerve agent) [129]. Molecular nanoarchitectonics, such as signaling units and spacers, are important in the design. Molecular nanoarchitectonics aimed at molecular recognition is important not only in chemosensing, but also in a wide range of applications such as supramolecular catalysis and supramolecular medicinal chemistry. Fukuhara and co-workers reported the fabrication of a bisporphyrin bisthiourea binaphthyl-conjugated chemosensor whose structure can be controlled using chiral dianions as effectors (Figure 4) [130]. The unique conjugated structure of this chemosensor was found to be capable of second excited-state fluorescence. The positive anisotropic allosterism amplified the binding constant of the sensing to the amino acid guest by a factor of 1000 over the reference compounds. It is noteworthy that the signal amplification by allosterism of the chemical sensor was obtained by well-considered molecular nanoarchitectonics.

**Figure 5:**
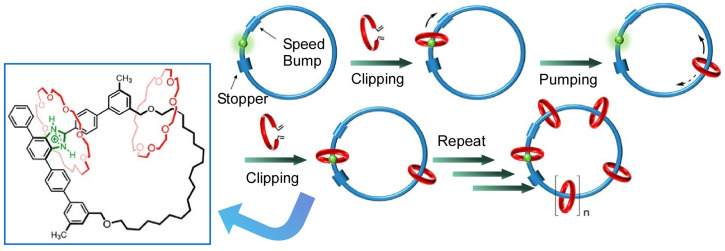
Controlled synthesis of radial [n]catenanes as molecular necklaces through clipping-followed-by-pumping. Reprinted with permission from Reference [131]. Copyright 2022 American Chemical Society.

As a molecular nanoarchitectonics approach conducive to molecular machine design, Zhang, Zhu, and co-workers demonstrated a controlled creation of radial [n]catenanes as molecular necklaces (Figure 5) [131]. Their synthetic tactic combines iterative synthesis with unidirectional molecular transport. This method can be thought of as clipping-followed-by-pumping. In the clipping step, a templated ring-closing reaction allows the introduction of acyclic precursors into specific recognition sites of large molecular loops. The pumping process then moves the wheel away from equilibrium and regenerates the recognition site so that a second clipping reaction can occur. Thereafter, the clipping and pumping process is repeated, resulting in the controlled synthesis of the molecular necklace.

**Figure 6:**
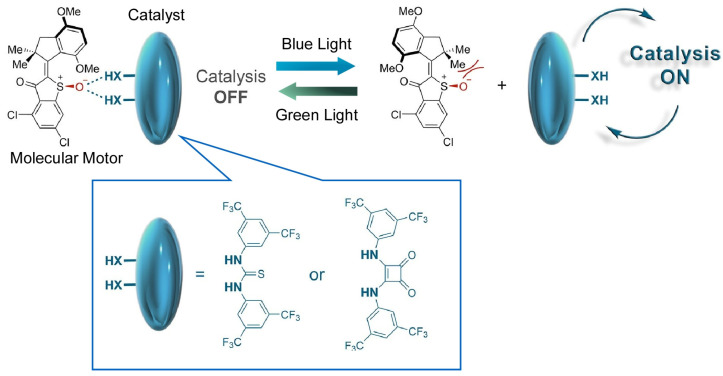
Light-induced shape changes of a hemithioindigo-based molecular motor to control the efficiency of a hydrogen-bonded organocatalysts. Reprinted with permission from Reference [132]. Copyright 2020 American Chemical Society.

Architecting individual molecular motors and molecular switch-like functions into larger metafunctional systems is an important step in the study of molecular machines. Grill and Dube relayed the light-induced shape changes of a hemithioindigo-based molecular motor to the catalytic efficiency of a chemical reaction [132]. They reported a modular nanoarchitectonics functional linkage (Figure 6). Rotational conformational changes in molecular motors can recognize hydrogen-bonded organocatalysts and control their catalytic activity. In the high-affinity molecular motor isomeric state, the organocatalysts are hydrogen-bonded via sulfoxide oxygen atoms, which inhibits their catalytic activity. Upon irradiation with visible light, the low-affinity molecular motor isomer is formed, the organocatalyst is released, and catalytic action is initiated. As described above, the dynamic and reversible remote control of catalytic reactions is demonstrated by the switching ability of molecular motors. This nanoarchitectonics approach also has the advantage of easy replacement of catalysts and tuning of reaction conditions because of the modularity of functional sites. Yamamoto, Suginome, and co-workers also reported the control of catalytic function through dynamic structural changes [133]. They demonstrated the use of a single-handed dynamic helical polymer as a nucleophilic catalyst in the asymmetric Steglich-type O-to-C aryloxycarbonyl rearrangement reaction of 3-substituted indole-2-yl aryl carbonates. Due to solvent effects, the polymer catalyst reversed the helix orientation and catalyzed the reaction giving the opposite enantiomer.

**Figure 7:**
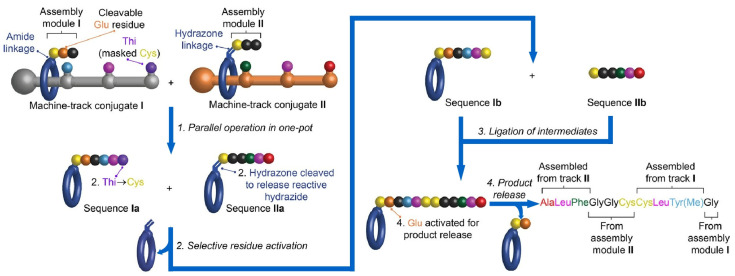
Molecular nanoarchitectonics of decapeptides by parallel manipulation of rotaxane-based molecular machines. Reprinted with permission from Reference [134]. Copyright 2021 American Chemical Society.

**Figure 8:**
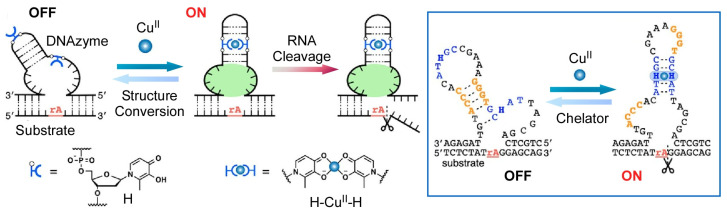
The control of DNA enzymes incorporating artificial hydroxypyridone ligand-type nucleotides (H) that form CuII-mediated base pairs. Reprinted with permission from Reference [135]. Copyright 2020 American Chemical Society.

Controlled synthesis of biomolecules by molecular machines has also been reported. Leigh and co-workers reported on the molecular nanoarchitectonics of decapeptides by parallel manipulation of rotaxane-based molecular machines [134]. First, two oligopeptides are synthesized simultaneously in the same reaction system as shown in Figure 7. Decapeptides are then synthesized through selective residue activation of oligopeptide intermediates, ligation, and product release. The synthesis of long oligopeptides by the parallel operation of these two molecular machines is a method reminiscent of protein ligation and post-translational modification in biological systems. It allows the synthesis of products that are beyond the capabilities of individual small molecule machines. As a nanoarchitectonics of molecular machine function using DNA, Shionoya and co-workers have demonstrated the control of DNA enzymes incorporating artificial hydroxypyridone ligand-type nucleotides (H) that form Cu^II^-mediated base pairs (H-Cu^II^-H) and logic gate control was reported (Figure 8) [135]. DNA enzymes with H-H pairing in the stem region are catalytically inactive in the absence of Cu^II^ ions. The formation of H-Cu^II^-H base pairing by Cu^II^ ions leads to an intrastrand conversion from the inactive to the active conformation, allowing allosteric control of DNA enzyme activity. Furthermore, by incorporating H-Cu^II^-H pairing into Ag^I^-dependent DNAzymes, they have developed a DNAzyme that exhibits an AND logic gate response to Cu^II^ and Ag^I^ ions. DNA nanoarchitectonics via metal-mediated artificial base pairing formation can realize molecular machines and logic gates.

**Figure 9:**
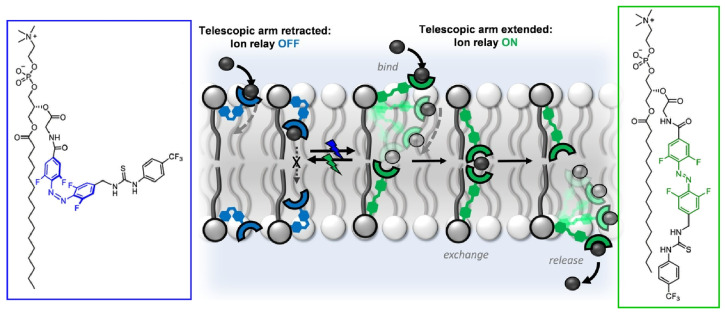
Artificial ion transporter on the opposite side of a lipid bilayer can serve as a molecular machine-like ion transport relay using a photo-responsive stretchable arm. Reproduced under terms of the CC-BY license [136].

Langton and co-workers demonstrated that an artificial ion transporter on the opposite side of a lipid bilayer can serve as a molecular machine-like ion transport relay (Figure 9) [136]. The transporter incorporates a photo-responsive stretchable arm that can be reversibly controlled in response to exposure to blue and green light. When the arms are extended by photoisomerization, anions can be passed between transporters on opposite sides of the membrane. This system functions as a collection of mechanical components that respond to stimuli, reminiscent of a robotic arm on a factory assembly line. The actions of these parts work in a coordinated manner to mediate ion transport. This is a molecular-level mechanical process assembly by nanoarchitectonics. Furthermore, by fixing, orienting, and controlling the relative positions of molecular machines, it will be possible to architect systems in which multiple components work in concert. Complex functions such as those of biological systems are also expected to be realized.

**Figure 10:**
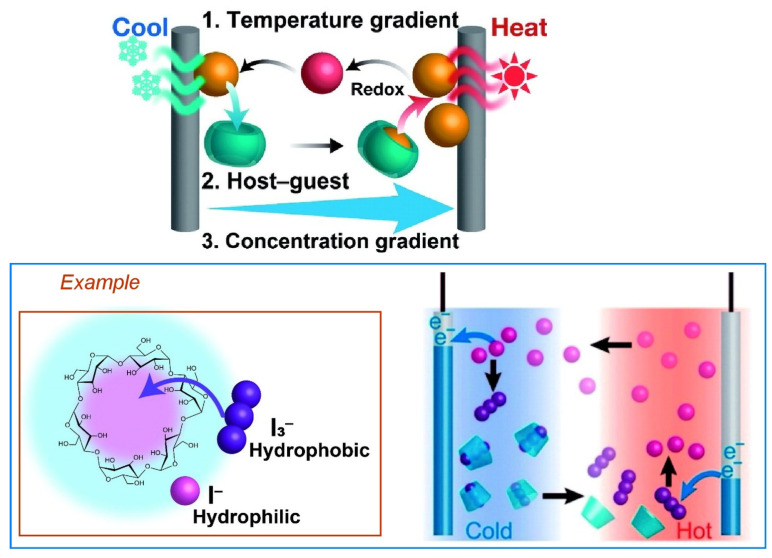
A supramolecular thermocell using thermo-responsive host-guest interactions to re-generate electrochemical energy from low-grade heat sources. Reprinted with permission from Reference [137]. Copyright 2021 Chemical Society of Japan.

Successful systemic relay of fundamental supramolecular phenomena such as molecular recognition can be used to architect energy-producing systems. Yamada and co-workers are developing a supramolecular thermocell that uses thermo-responsive host-guest interactions to regenerate electrochemical energy from low-grade heat sources [137]. Figure 10 shows the mechanism of operation of a supramolecular thermocell in which the host molecules transport guest ions from the cooling electrode to the heating electrode. First, a temperature difference is applied between the two electrodes. Guest ions on the cooling side are captured and released by the host molecules to the heating side. The concentration gradient of the guest ions generates a potential difference, which increases the electromotive force. Host molecules that lose their guests diffuse back to the cooling side. As a result, the redox species carried by the host molecules selectively move from the cooled electrode to the heated electrode, creating a concentration gradient of guest redox species and providing an additional voltage to the thermocouple. The elements required for nanoarchitectonics in supramolecular thermocells include high selectivity of the host molecule to capture oxidizing or reducing species as guests, suppression of redox activity after formation of the host-guest complex, and large entropy change upon release of the guest molecule in response to temperature change.

### 2.2. Micromachines and Microrobots

Micromachines and microrobots, whose sizes are several orders of magnitude different from molecular machines, are also regarded as attractive research targets. In addition to the use of nanoparticles and microparticles and their modification with functional molecules and polymers, a variety of technologies are used to fabricate structures, such as material processing by microfabrication. This technology-intensive aspect is common to the development of functional materials through nanoarchitectonics. In contrast to molecular machines and nanomachines, which are fundamental and at the scientific frontier, micromachines, with their advanced fabrication technologies, have many examples of research focused on practical aspects, such as medical and environmental applications. Some micromachines are self-propelled and are often referred to as microrobots. It is impossible to comprehensively follow all such research, so this review will pick up examples from recent years to illustrate their diversity as follows. First, it will discuss the materials and functions used to form functional microstructures and their analysis methods, followed by examples of microrobots with a strong application orientation.

As seen in the mechanical devices such as actuators, the function and movement of micromachines are often discussed in correlation with changes in their constituent molecules. Hayashi proposed a simple but detailed method to study nanoscale structural changes and macroscopic crystal deformation in flexible organic crystals [138]. Stress was applied to flexible organic crystals and then relaxed the stress to induce reversible macroscopic crystal deformation. The method was demonstrated to analyze the reversible nanoscale structural unit cell changes in the crystal structure due to bending stress and relaxation by X-ray diffraction using a curved stage fixture. Reversible structural changes can be quantitatively measured without using a synchrotron X-ray analyzer. Among the examples of those analyzed, an unusual effect, the negative Poisson effect, was observed. The method may facilitate understanding of dynamic changes in nanostructural unit vesicles in flexible organic crystals that can also lead to micromachines. As a translator of molecular structure into information, control of the helical structure of polymers can lead to the transfer of chiral information and memory functions. Yashima and Maeda summarize in a recent paper synthetic helical polymers with dynamic and static memory of helicity [139]. In particular, they emphasize the tactic of helical structure induction and the associated dynamic and static memory. After polymerization of achiral or racemic monomers, noncovalent chiral interactions with nonracemic guests can generate helix sense-selective right- and left-handed helical polymers. No specific chiral monomers or chiral catalysts or initiators are required for the synthesis of helical polymers. In addition to the chiral memory functionality, the polymer is expected to be used in organic spintronics devices, enantioseparation in helical cavities, and asymmetric catalysis.

**Figure 11:**
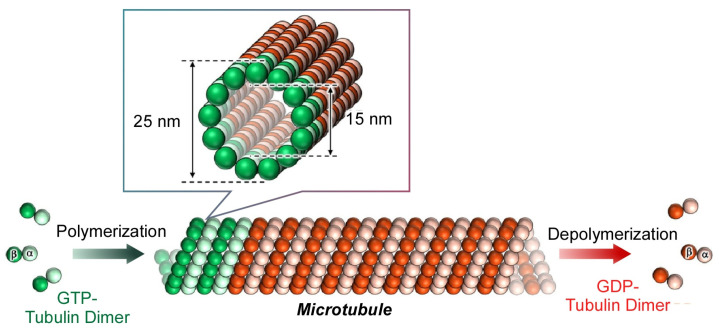
Biological microtubules formed through dynamic polymerization and depolymerization of tubulin dimers. Reprinted with permission from Reference [140]. Copyright 2021 Chemical Society of Japan.

**Figure 12:**
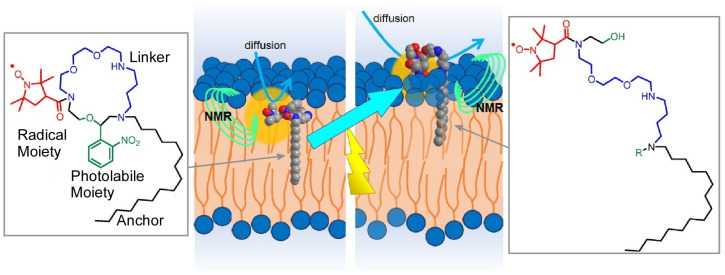
A photodegradable macrocyclic compound to measure water around liposomes in situ that is originally buried in the lipid bilayer and then brought out by the photocleavage reaction. Reprinted with permission from Reference [152]. Copyright 2022 Chemical Society of Japan.

Some biomaterials can function as micromachines, and there have been attempts to use them artificially. Inaba and Matsuura summarized the use of microtubules in a recent review (Figure 11) [140]. Microtubules are an important part of the cytoskeleton. Various proteins have been found to exist inside microtubules, and the interior of microtubules is also attracting attention as an artificial reaction field. Physical properties and functions of microtubules, such as length, stiffness, stability, and functional speed, can be tuned by encapsulation with peptides. Genetic modification of tubulin is another method of introducing molecules into microtubules in living cells. It has also been shown that microtubules can be modified by encapsulating artificial molecules. The use of motor proteins such as kinesins that walk in one direction on microtubules has also been devised [141,142]. This is used to transport objects and control the movement of microtubules. DNA micro-machines based on DNA origami method [143,144,145], DNA nanotechnology [146,147,148], and DNA nanoarchitectonics [149,150] have also been studied extensively, although the details are beyond the scope of this article, and DNA sequencing-specific nanoarchitectonics can be used to express a variety of functions. For example, a recent review by Kim and co-workers et al. describes molecular systems based on single nucleotide polymorphisms, their fluorescent behavior in duplex DNA, the effect of fluorophore labeling on the fluorescent signal of modified oligonucleotides, and pH-responsive nucleic acid-modified fluorescent biosensors [151]. In addition, how the artificial probe molecule interacts with the cell or its model is also important to analyze its function for the machine. Kageyama and co-workers used dynamic nuclear spin polarization, which increases the sensitivity of nuclear magnetic resonance by enhancing the magnetization of nuclear spins with that of electron spins [152]. In their study, they used a photodegradable macrocyclic compound to measure water around liposomes in situ and site-selectively, elucidating that the radical moiety of the probe molecule, originally buried in the lipid bilayer, was brought out by the photocleavage reaction of the macrocyclic compound, as shown in Figure 12.

**Figure 13:**
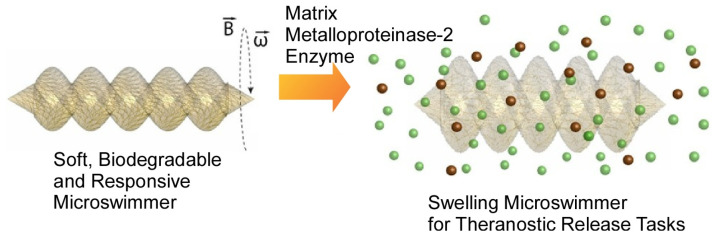
Hydrogel-based, hand-created, magnetically driven and controlled, enzymatically degradable miroswimmers with a swelling process for releasing the loaded drug molecules. Reproduced under terms of the CC-BY license [157].

Nanocarbons and inorganic nanoparticles are also used as materials for nanoarchitectonics of micro-level functional materials [153,154,155]. For example, Imae, Tsutsumiuchi, Kawai, and co-workers nanoarchitectonized a composite consisting of magnetite nanoparticles, carbon dots, and carbon nanohorns [156]. The loading and release of drugs (doxorubicin and gemcitabine) were investigated using these composites. It was found that there is a photodynamic/photothermal effect under laser emission on the drug release. The development of nanoarchitectonics microrobots of inorganic materials has also been reported. Sitti and co-workers reported on a hydrogel-based, hand-created, magnetically driven and controlled, enzymatically degradable microswimmers, which are enzymatically degradable (Figure 13) [157]. The microswimmer was fabricated by 3D printing from a suspension of magnetic precursors consisting of gelatin methacryloyl and superparamagnetic iron oxide nanoparticles. The microswimmers respond to pathological markers in the microenvironment for theranostic drug delivery and release. At normal physiological concentrations, the microswimmer was completely degraded by matrix metalloproteinase-2 enzyme in 118 h. The degradation results in soluble, non-toxic products. The microswimmer reacts rapidly to pathological concentrations of matrix metalloproteinase-2 enzyme and undergoes a swelling process, releasing the loaded drug molecules.

**Figure 14:**
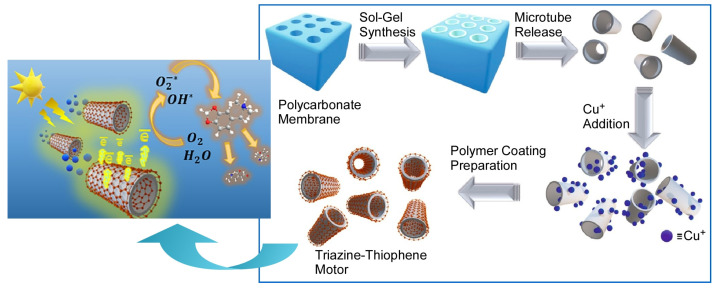
A tubular inorganic/organic hybrid that combines mesoporous silica with active polymers containing thiophene and triazine units as photoactive microrobots for efficient directional motion under fuel-free conditions. Reprinted with permission from Reference [158]. Copyright 2021 American Chemical Society.

**Figure 15:**
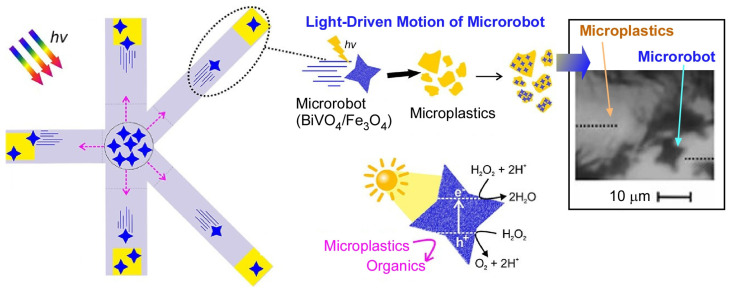
Microrobots capable of moving by visible light and capturing/degrading microplastics in a complex multichannel maze. Reprinted with permission from Reference [159]. Copyright 2021 American Chemical Society.

Light-driven microrobots based on organic semiconductors have attracted much attention in the past few years. Pumera and co-workers have developed a tubular inorganic/organic hybrid that combines mesoporous silica with active polymers containing thiophene and triazine units as photoactive microrobots (Figure 14) [158]. The microrobots have a well-defined tubular structure, which allows for efficient directional motion under fuel-free conditions. It was also observed that the microrobot can perform rising and rotating motions depending on the amount of polymer coating. As a function of this microrobot, the group succeeded in capturing and decomposing harmful psychoactive substances contained in wastewater, such as methamphetamine derivatives. They also developed a microrobot that can photodegrade microplastics in a confined space. The potential risks of microplastics to humans and marine systems are threatening. They developed a microrobot that can be moved by visible light and can capture and degrade microplastics in a complex multichannel maze (Figure 15) [159]. The microrobots are hybridized with a built-in photocatalyst (BiVO_4_) and magnetic material (Fe_3_O_4_). These microrobots are self-propelled under sunlight and can be moved precisely by magnetic fields in microchannels. The localized self-stirring effect at the nanoscale allows them to efficiently degrade a variety of synthetic microplastics such as polylactic acid and polycaprolactone. In other words, this photocatalytic microrobot does not require a mechanical stirring device, which is typically required.

**Figure 16:**
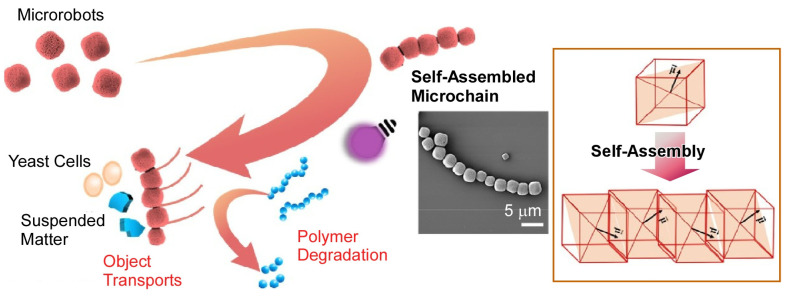
Shape-controlled microrobots to form chain-like aggregate structures. Reprinted with permission from Reference [160]. Copyright 2022 American Chemical Society.

As in many examples in nature, there are many functional units that work collectively and in concert to accomplish complex tasks. Some attempts have been made to group microrobots. Pumera and co-workers reported that shape-controlled microrobots form chain-like aggregate structures (Figure 16) [160]. Unlike microrobots of other shapes, the cubic hematite/platinum microrobots self-assembled into ordered microchains because of asymmetric magnetic dipole moments in the crystal. The assembled microchains exhibited different synchronized motions under light irradiation, depending on the mutual orientation of each microrobot during assembly. They can pick up, capture, and transport microobjects such as yeast cells and suspended solids in water. They can also perform multiple tasks such as degrading polymeric materials. Modification of microrobots with other polymers has also been done. Pumera and co-workers designed a magnetic microrobot that combines biocompatible polymers (polycaprolactam and polyethyleneimine) with Fe_3_O_4_ magnetic nanoparticles [161]. This microrobot was used to efficiently remove nerve agents from contaminated water. It was able to remove about 60% of nerve agent from the water sample in a short time. This example shows that the microrobot may be useful for large-scale water treatment for environmental remediation.

**Figure 17:**
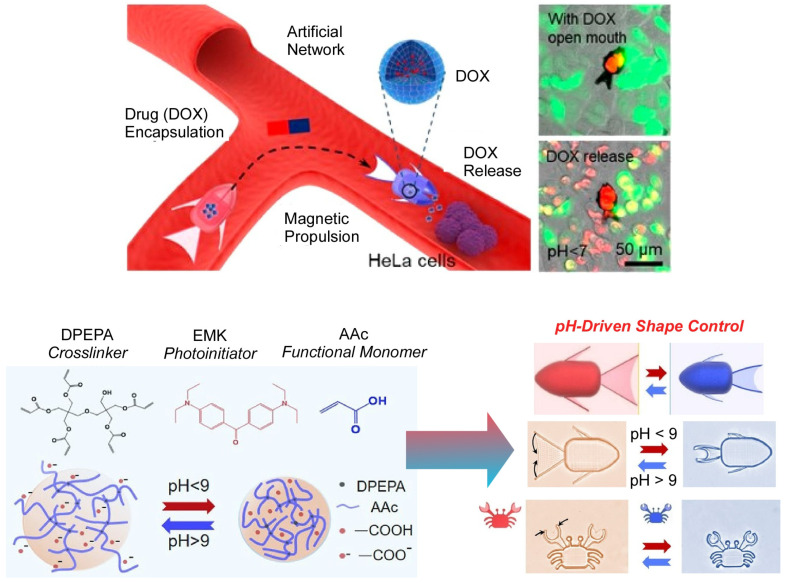
Environmentally adaptive shape-shifting microrobots for targeted delivery of microparticles through grasping, transport, and release. Reprinted with permission from Reference [162]. Copyright 2021 American Chemical Society.

The ability to change its shape and size is also a useful property in the design of highly functional microrobots. In particular, more complex drug delivery operations such as drug encapsulation and release in practical biological applications also require that microrobots have shape adaptability to dynamic environments. Li, Zhang, Wu, and co-workers developed an environmentally adaptive shape-shifting micro architectural means to programmatically code different expansion rates into a pH-responsive hydrogel for robot development (Figure 17) [162]. Combined with magnetic propulsion, the microcrab, a shape-deformable crab-like micro-robot, can perform targeted delivery of microparticles through grasping, transport, and release by opening and closing its claws. In addition, microfish, a goldfish-shaped microrobot, can open and close its mouth to achieve localized processing of HeLa cells within an artificial vascular network. It is expected that the introduction of imaging techniques to optimize the size and motion control of these microrobots will enable more practical and complex micro-drug delivery operations and on-demand drug release.

## 3. Working on Water

### 3.1. Molecular Machines at the Air-Water Interface

The role of interfaces in molecular machine and microrobotics research is significant. In particular, direct observation and evaluation of nanomachines such as molecular machines and nanocars are now being performed in detail on solid substrates. Although it is difficult to conduct advanced research such as observation at the molecular level at the liquid interface, which is a softer and more fluctuating environment as an interface, it has the potential to bring out the dynamic properties of molecular machines. In particular, the interface with water (air-water interface), which is common to biological systems, can be a new frontier for the study of molecular machines.

The air-water interface is also known as a field where excellent molecular recognition properties are achieved through hydrogen bonding, despite the presence of water [163]. Hydrogen bonding is due to charge bias through hydrogen atoms. Therefore, hydrogen bonds are difficult to form in media with high dielectric constants. On the other hand, the medium of biological systems is water. The key to understanding this contradictory fact lies at the interface. Molecular recognition in biological systems rarely occurs in bulk solution. It takes place in a broadly defined interfacial environment, for example, on the surface of a cell membrane, inside an enzyme pocket, or on an aligned macromolecular chain, such as DNA. Using more systematic analytical methods, Kunitake, Kurihara, Ariga, and the others have quantitatively demonstrated that the recognition system of biomolecules by hydrogen bonding at the air-water interface is widely achieved for objects such as nucleobases, nucleotides, sugars, amino acids, and peptides [164,165,166]. Another important role of the interface has recently been proposed as a field that connects macroscopic phenomena with molecular and nano phenomena. In other words, it is at the interface that we can manipulate molecules with hand-motion-like macroscopic mechanical actions, e.g., driving molecular machines and tuning molecular receptor structures with hand movements [167,168]. At a two-dimensional interface, lateral changes in magnitude can be triggered with visible size. On the other hand, in the perpendicular direction, nano- and molecular-scale changes can be induced. The interface is a place where phenomena of different magnitudes can be coupled. Driving molecular machines and optimizing the structure of receptor molecules can be done at the air-water interface with macroscopic mechanical actions. This is a new concept of hand-operating nanotechnology [169], which is to manipulate molecular structures with a hand-like motion.

**Figure 18:**
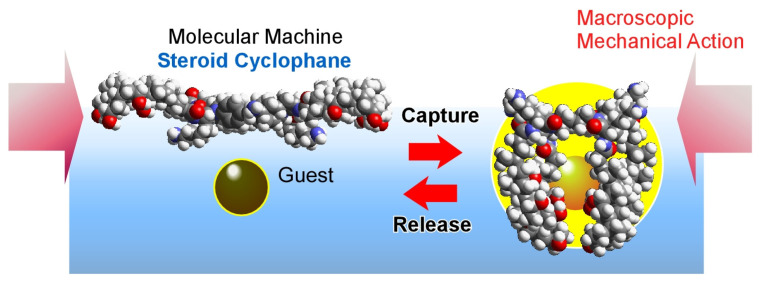
Manual actuation of a molecular machine using the steroid cyclophane at the air-water interface for reversible capture and release of a guest molecule.

Manual actuation of a molecular machine using the steroid cyclophane at the air-water interface was investigated (Figure 18) [170,171]. This molecular machine has a cyclophane ring structure in the center, to which four steroidal moieties are introduced via flexible spacers. In particular, the steroid moiety is a cholic acid unit with three hydroxyl groups on one side. This molecular machine takes on an open conformation, attaching the hydrophilic side of the cholic acid to the water surface if no pressure is applied. When pressure is applied by macroscopic compression of the monolayer, this molecular machine forms a three-dimensional cavity. In this process, it captures a guest molecule in the water as if it were grasping something with its hand. When a naphthalene-type fluorescent molecule is used as a guest, a marked increase in fluorescence intensity is observed around 2 nm^2^ of molecular occupied area. It was found that the steroidal cyclophane molecules formed a three-dimensional cavity and grabbed the guest molecules present in the water. Macroscopic repetition of compression and expansion of the monolayer at the level of tens of centimeters was found to cause a corresponding repetitive catch and release of the guest molecules. Using the molecular machine at the air-water interfacial environment, a bulk mechanical stimulus (visible size, tens of cm) that can be moved by hand can grab and release molecules that are at the nanoscale.

A more delicate structural control is the mechanical tuning of molecular receptors. For example, the molecular receptor cholesterol-armed cyclen is a molecule that can produce a chiral twist, and its chiral environment allows it to selectively adsorb chiral molecules [172]. This molecular receptor can be lined up at the air-water interface and the chiral twist can be changed by macroscopic compression to gradually change the chiral environment of the two-dimensional surface facing the water. Depending on the surface pressure of the compressing surface, the selectivity of the optical isomer bonding of amino acids was successfully reversed. It is now possible to identify chirality by hand-motion-like macroscopic actions. In another molecular receptor example, a molecular receptor molecule, armed cyclononane, was arranged as a monolayer on the water surface and pressure was gradually applied across the membrane [173]. Structural tuning showed that uracil could be selectively discriminated with 64-fold accuracy under optimal conditions. DNA and RNA both use adenine as the complementary base, and these natural nucleic acids cannot discriminate between thymine and uracil. In the above example, the membrane was mechanically pressed to achieve selective recognition of nucleobases, which is not possible with DNA or RNA.

**Figure 19:**
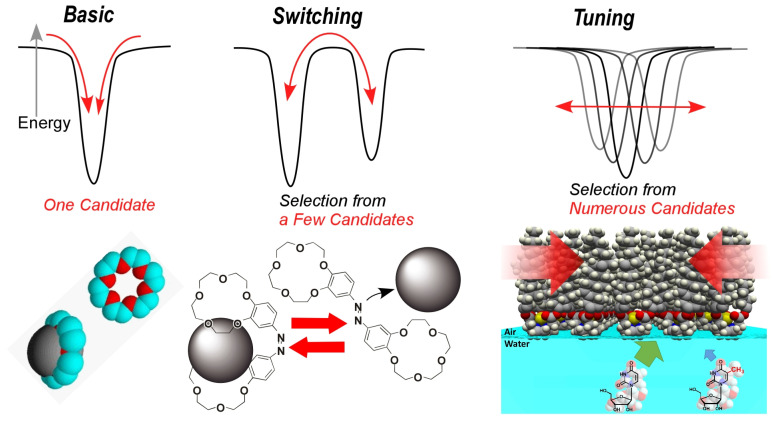
Summary the modes of molecular recognition: basic, switching, and tuning.

The above examples change the way we think about molecular recognition. Figure 19 summarizes the modes of molecular recognition [174,175]. Molecular recognition began with the primary recognition of guests by host molecules such as crown ethers and cyclodextrins [176,177,178]. A mode of switching selectivity (transitioning between multiple stable states) was incorporated by isomerization of the host [179,180]. In fact, most of the current molecular machines are based on this switching of stable states and have not departed from this stage [181,182]. On the other hand, the above mechanical method at the air-water interface, which tunes between countless states to find the optimal structure, opens a new page in the mode of molecular recognition. Organic molecules are characterized most by their softness, and to think of them uniquely in a crystal-like structure or in only a few states is not a legitimate use of the properties of organic materials. When organic molecules are considered as machines, their structures can be continuously changed, and their potential abilities can be brought out by tuning their functional properties [183]. For this purpose, it is necessary to tune the molecular structure by mechanical and other methods, and an effective method is to link macroscopic behavior to molecular behavior in an interfacial environment.

**Figure 20:**
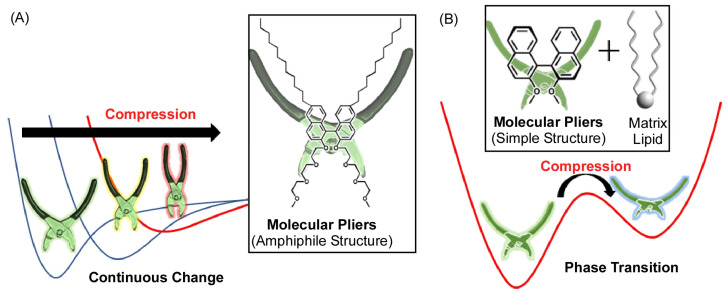
Controls of binaphthyl-type molecular pliers at the air-water interface: (**A**) continues change; (**B**) phase-transition-based digital change.

In addition, a simpler molecular machine was used to quantitatively analyze molecular deformation at the interface (Figure 20A) [184]. Here, binaphthyl-type amphiphiles are used as openable molecular pincers to investigate the extent to which macroscopic mechanical energy is used for molecular deformation. First, the molecular pliers were assembled at the air-water interface and gradually compressed, and the value of the dihedral angle of the binaphthyl group (the degree to which the molecular pliers are closed) was measured. Based on this data, the energy required for molecular deformation was calculated by quantum chemical calculations and other methods. The energy values for the mechanical deformation of macroscopic monolayers were also estimated thermodynamically. When both were compared, the values were very similar. When the surface pressure increases from 0 to 10 mN m^−1^, about 0.2 kcal mol^−1^ of mechanical energy is stored, and about the same value of molecular deformation energy is used. At higher pressures, the molecular deformation energy is smaller than the mechanical energy, but the results suggest that macroscopic mechanical energy can be used very efficiently to deform molecular machines in interfacial systems.

Molecules with hydrophilic and hydrophobic portions have been studied as molecular pliers, and it was clear that the dihedral angle changes continuously and analogously in response to mechanical stimuli applied externally. However, it was a molecular deformation with unchanged helicity, cisoid-to-cisoid. Using a system of simpler, more crystalline binaphthyl molecules dispersed in a matrix, a cisoid-to-transoid (closed-to-open) asymmetric control with a change in helicity (right-handed to left-handed or vice versa) was successfully (Figure 20B) [185]. This is based on the fact that binaphthyl molecules dissolved in the matrix lipid in a cisoid form at low pressure precipitate as crystals of the transoid form from the two-dimensional matrix with increasing pressure. This transformation can be reversible by repeated macroscopic compression and expansion movements into the monolayer. In this case, the biplane angle does not change continuously in an analog manner, but digitally between the two values. This is probably due to the use of highly crystalline molecular elements and the increase in the number of phases that can be taken thermodynamically due to the increase in the number of components.

Żywociński, Hołyst, and co-workers measured the frequency of collective molecular precession as a function of temperature in ferroelectric liquid crystal monolayers at the air-water interface [186]. This precession is driven by the unidirectional flux of water molecules evaporating through the interface. The collective rotation of monolayers is 9-14 orders of magnitude slower than that of single molecules. The angular velocity is zero as the monolayer angles into the solid phase. The energy of the torque that drives the rotation per molecule is seven orders of magnitude less than the thermal energy. Nevertheless, the rotation is very stable. This stabilizing effect is probably due to the collective motion of a large number of nano windmills in concert on the millimeter scale. Thus, such a system could be a candidate for a reliable artificial molecular engine, despite its small energy density per molecular volume. The collective behavior of molecular machines integrated at the air-water interface may be reflected in macroscopic properties such as surface pressure. For example, Gengler, Feringa, and co-workers examined the behavior at the air-water interface of a bis(thiaxanthylidene)-based photo-switching amphiphile as an amphiphilic compound featuring molecular motor motion [187]. It was found that the central core of this amphiphilic molecule changes from an antifolded to a co-folded form upon photoisomerization, resulting in a change in surface pressure in either direction. Furthermore, by mixing this photoisomerized amphiphile with the phospholipid dipalmitoylphosphatidylcholine, the surface pressure response properties of the monolayer could be modulated. Although this is an approach in the opposite direction to the aforementioned method of manipulating molecular machines by hand, the concept of connecting the movement of the molecular machine and macroscopic (mechanical) properties at a dynamic interface is common.

**Figure 21:**
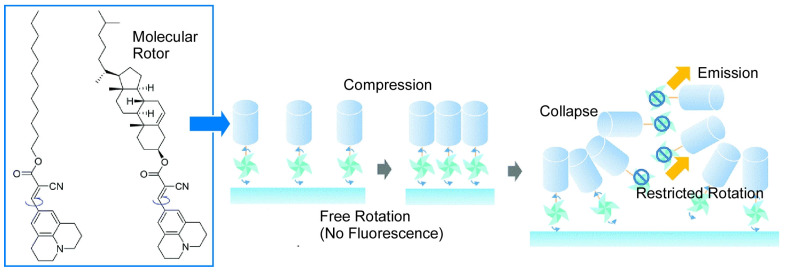
Rotational behavior of a twisted intramolecular charge trans-fer-type 9-(2-carboxy-2-cyanovinyl)julolidine derivative as a molecular rotor at the air-water interface. Reprinted with permission from Reference [188]. Copyright 2018 Royal Society of Chemistry.

The behavior of molecular machines such as molecular rotors at the air-water interface and in thin films is affected by the surrounding environment, such as coexisting monolayer materials and surface pressure. Mori et al. investigated the rotational behavior of a twisted intramolecular charge transfer-type 9-(2-carboxy-2-cyanovinyl)julolidine derivative as a molecular rotor by in situ fluorescence spectroscopy (Figure 21) [188]. Monomer emission was suppressed in monolayers, suggesting that intramolecular rotation is not suppressed even in densely ordered monolayers. The molecular rotors have linear alkyl or cholesteryl groups as hydrophobic fields, and the free volume of rotation is considered to be secured in monolayers packed with these hydrophobic parts. The rotation of the molecular rotor was maintained even when this monolayer was stacked as a multilayer LB film while maintaining order. Conversely, when the ordered two-dimensional structure was disrupted by collapsing the monolayer, the rotation of the molecular rotor was inhibited. Even when molecules were packed into an area equal to the size of the molecule, intramolecular rotation of the molecular machine was not inhibited if the molecules were aligned two-dimensionally. This finding is a good guide for fabricating active molecular machines as membrane structures on substrates. Mori et al. similarly investigated the rotational behavior of a molecular rotor with a different molecular diameter, 4-farnesyloxyphenyl-4,4-difluoro-4-bora-3a,4a-diaza-s-indacene (BODIPY-ISO) [189]. In the case of this molecular rotor, it was found that it can be controlled according to local condensation conditions in monolayers at the air-water interface and in mixed self-assembled monolayers.

Saito and co-workers have developed a series of hybrid π-conjugated molecules that combine the advantages of rigidity with flexibility, based on the viewpoint of moving the π-conjugated backbone. The result is a series of hybrid π-conjugated molecules that have the advantage of flexibility while taking advantage of rigidity. These molecules are named as FLAP (flexible aromatic photofunctional systems) [190,191]. Nakanishi and co-workers investigated the behavior of polarity-independent FLAP molecules with hydrophobic/hydrophilic substituents at the air-water interface [192]. Fluorescence emission analysis revealed that the flattening dynamics in the excited state is restricted at the air-water interface. As can be seen from this example and the behavior of molecular rotors described above, the behavior of molecular machines at the air-water interface environment can be controlled in a variety of ways by differences in molecular structure and molecular motion environment.

**Figure 22:**
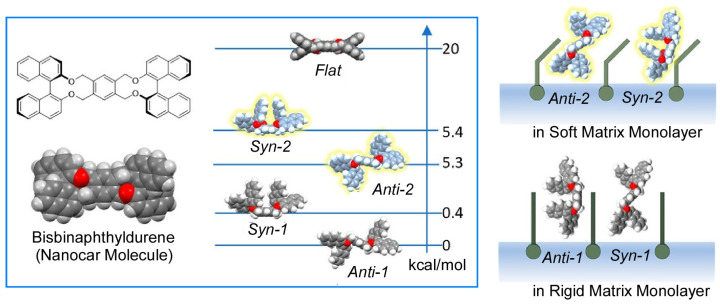
Behavior of bisbinaphthyldurene molecules (considered as nanocar molecules on a solid surface) at the air-water interface. Reprinted with permission from Reference [194]. Copyright 2020 American Chemical Society.

Comparing the same molecule or molecular machine in various interfacial environments is also an interesting research target. The behavior of bisbinaphthyldurene molecules, considered as nanocar molecules on a solid surface under ultrahigh vacuum and cryogenic conditions [193], was investigated at the air-water interface (Figure 22) [194]. Bisbinaphthyl durene molecules can form multiple conformers by joining two binaphthyl groups via a central durene moiety. Density functional theory calculations have shown that the bis-binaphthyl durene molecule can have five conformers: anti-1, anti-2, syn-1, syn-2, and flat. On the gold surface, the system can have different conformers (syn dimer and flat) and the other two (anti-1 and syn-1) in solution. At the air-water interface, the conformational ratio changes depending on the environment of the mixed monolayer. In rigid mixed monolayers with low miscibility with bisbinaphthyldurene molecules, the bisbinaphthyldurene molecules self-aggregate to form stable anti-1 and syn-1 conformations. Conversely, dispersed in soft lipids that are highly miscible with bisbinaphthyl durene molecules, the unstable anti-2 and syn-2 conformations are dominant. At the air-water interface, it is possible to control the conformation of the target molecule depending on the environment, such as the state of miscibility with the mixed lipid. Similarly, the aggregation state of monobinaphthyl durene molecules was controlled by applying different mechanical stimuli at the air-water interface [195]. When the vortex LB method [196,197] is applied to the water surface, the molecular aggregation state and conformation of the monobinaphthyl durene molecules depend on the molecular density and vortex flow velocity at the interface.

**Figure 23:**
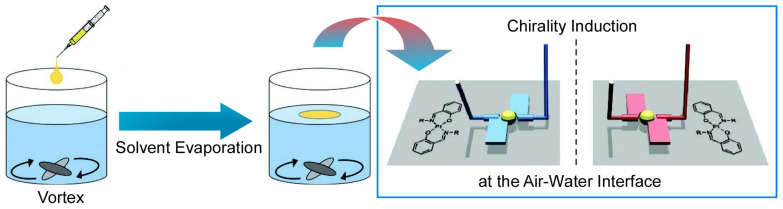
Control of the chirality of trans-bis(salicylaldiminato)platinum(II) complexes by the direction of the vortex flow at the air-water interface. Reprinted with permission from Reference [198]. Copyright 2022 Wiley-VCH.

Naota and co-workers have very recently studied the chirality and magnitude of circularly polarized luminescence of trans-bis(salicylaldiminato)platinum(II) complexes by using a mechanical rotational stimulation method at the air-water interface [198]. The chirality and size of the circularly polarized emission of trans-bis(salicylaldiminato)platinum(II) complexes can be precisely controlled by the size and direction of the vortex flow at the air-water interface (Figure 23). The supramolecular chirality of aggregates composed of trans-bis(salicylaldiminato)platinum(II) complexes with achiral hexadecyl chains form achiral amorphous materials with nonchiroptic properties under non-eddy current conditions. Conversion of the direction of the vortex flow applied to the air-water interface was confirmed to result in aggregates exhibiting chiral properties. The direction and size of the circularly polarized luminescence of the platinum(II) complex aggregates can be precisely tuned by the vortex conditions such as directions of rotation and flow velocity. In this case, an enhancement of vortex-induced luminescence was observed when the vortex flow velocity was increased.

**Figure 24:**
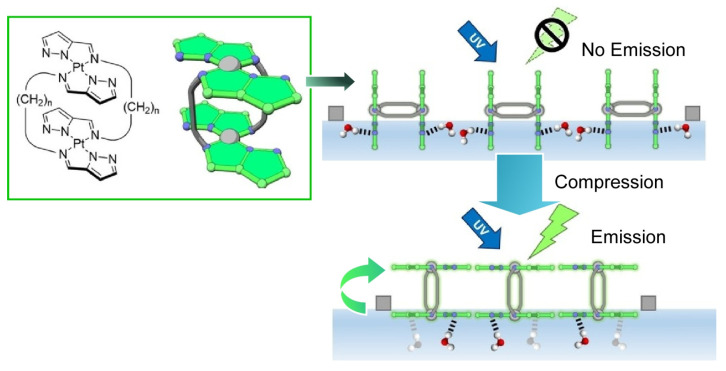
Submarine emission in which a double-paddle platinum complex is used to control the luminescence of cyclic amphiphilic molecules at the air-water interface by means of mechanical manipulation. Reprinted with permission from Reference [199]. Copyright 2019 Wiley-VCH.

Adachi et al. developed a new principle of “submarine emission,” in which a double-paddle platinum complex is used to control the luminescence of cyclic amphiphilic molecules at the air-water interface by means of mechanical manipulation (Figure 24) [199]. When a monolayer of double-paddle Pt complexes is formed at the air-water interface and the water surface is compressed from both ends, the luminescence intensity increases rapidly from a certain compression state. The orientation of the double-paddle Pt complex at the air-water interface changes from perpendicular to parallel as the monolayer undergoes a phase transition. Under low surface pressure, two co-planar coordination paddles stand independently on the water surface. The coordination paddles are partially immersed in the aqueous phase. Fluorescence emission is small in this case. Under high surface pressure, when the monolayer is compressed, it takes on another stable orientation, with one coordination face submerged in the aqueous subphase and the other virtually out of contact with the water in the air phase. The emission intensity is enhanced when the emitting site of the double-paddle Pt complex “floats” from the aqueous phase, a high dielectric medium, to the gas phase, a low dielectric medium. The submarine-like control of optical properties by molecular manipulation in a nanometer-sized asymmetric interfacial environment has been named the submarine emission mechanism. Furthermore, the related molecular systems have been systematically analyzed the behavior of such complex molecules at the air-water interface and proposed the concept of coordination amphiphilicity [200]. Molecular modeling and analysis of the aggregation behavior of the compounds suggest that the appropriate hydrophilicity of the coordination surface and intermolecular interactions, including hydrogen bonding, are important factors for monolayer formation.

### 3.2. Microrobots at the Air-Water Interface

As seen in the above examples, the air-water interface is a field where the dielectric constant varies greatly at the molecular level, with properties such as an overwhelming difference in magnitude between the film surface direction and the film thickness pressure direction, high fluidity and dynamism. Placing molecular machines, which are molecular-level objects, at these interfaces generates various scientifically innovative research. On the other hand, the molecular size properties of the air-water interface might not have a significant influence on larger microrobots. However, the surface of water is a place where molecular robots can move freely, and a variety of research is being conducted. In particular, many of the studies on microrobots are aimed at practical applications in aqueous environments. Here, the following part lists a few examples to illustrate the diversity of their functions at the air-water interface.

**Figure 25:**
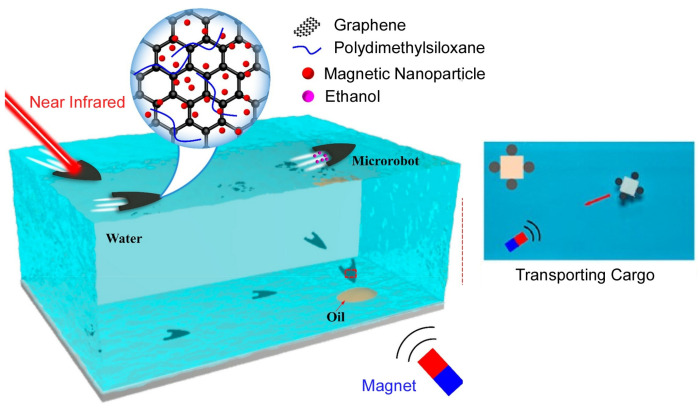
Multi-stimulus-responsive superhydrophobic microrobots that can drift at high speed on water through optical, magnetic, and chemical controls. Reprinted with permission from Reference [201]. Copyright 2022 American Chemical Society.

Superhydrophobic microrobots can swim efficiently and rapidly on water by external stimuli and have attracted great research interest for various applications. Jiao, Liu, and coworkers have developed a multi-stimulus-responsive superhydrophobic microrobot that can drift at high speed on water through optical, magnetic, and chemical controls (Figure 25) [201]. This multi-stimuli-responsive superhydrophobic microrobot is architected from graphene and magnetic nanoparticles. It can swim at high speed on and in water under the action of various stimuli such as magnets, infrared lasers, and ethanol molecules. The action of light and magnetic fields enables the multi-responsive microrobots to achieve coordinated motion. Additionally, complex motions such as circular, helical, and spiral motions can be realized by the combined action of chemical and magnetic field stimuli. As tasks, a wide range of multifunctional applications were explored, such as transporting objects, collecting spilled oil, and mixing solutions.

The development of hybrid microrobots capable of both terrestrial and underwater activities requires harmonizing and reconciling different behaviors and various constraints in different environments. Chen, Wood, and co-workers have developed a quadruped microrobot that can walk on land, swim in water, and transition between the two [202]. The microrobot uses a combination of surface tension and buoyancy to support its body weight. It can also move forward and turn while swimming by using passive flaps to generate differential drag. To operate in these diverse environments, it is necessary to consider devices for buoyancy control, leg structures that generate lift in addition to thrust, development of walking mechanisms, and stability in water. Wang, Yang, and co-workers reported a novel star-shaped magnetic microrobot that is capable of locomotion on the water surface and manipulation of microobjects [203]. The microrobot consists of a 40-μm-thick star-shaped sheet. It is activated by magnetic fields that are tilted and rotated with an unbalanced force acting on the microrobot from the water surface, allowing it to move along the interface. In addition to free movement on the underwater surface, it can capture, transport, and release non-magnetic microobjects by adjusting the frequency and direction of the rotating magnetic fields. Transport along specific trajectories and the assembly of multiple microballs have also been demonstrated. Because of its agility, ease of fabrication, and low cost, it is expected to be applied to micropart assembly, water pollution monitoring, or the elucidation of various interfacial phenomena.

Light-responsive superhydrophobic microrobots based on the Marangoni effect have attracted much attention, and Jiao, Liu, and co-workers have fabricated graphene/polydimethylsiloxane composite materials that exhibit excellent superhydrophobicity and photothermal properties [204]. Using the developed material, they fabricated microrobots of different shapes with superhydrophobicity and photothermal properties. The microrobots exhibit various motions such as linear, rotational, and oscillatory motions by the Marangoni effect when irradiated with infrared light. By changing the infrared irradiation area and the structure of the microrobot, the characteristics of the microrobot’s motion at the air-water interface can be changed. The water strider-type microrobot can glide on the water surface and make a 180^o^ rotational jump. These multiple capabilities overcome the disadvantages of microrobots with a single mode of locomotion.

**Figure 26:**
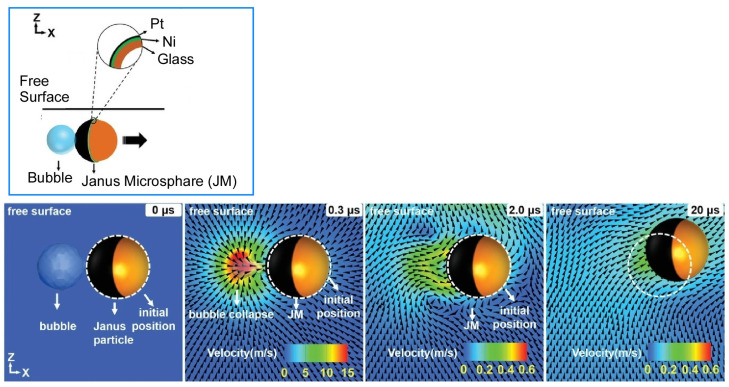
The bubble microrobot consists of hollow Janus microspheres controlled by a magnetic field with switching between working modes: pusher, gripper, anchor, and sweeper through operating mechanism of collapse of bubbles and the control of induced jet flow through the air-liquid interface. Reprinted with permission from Reference [205]. Copyright 2022 Wiley-VCH.

Zheng, Cui, and co-workers have developed a remotely operated bubble microrobot with multiple modes of operation and high maneuverability at the air-liquid interface (Figure 26) [205]. The bubble microrobot consists of hollow Janus microspheres controlled by a magnetic field and can switch between working modes: pusher, gripper, anchor, and sweeper. The operating mechanism of the bubble microrobot is the collapse of bubbles and the control of induced jet flow through the air-liquid interface. The collapse of the microbubbles and the accompanying directional jet flow enable these work modes. A vertical magnetic field was used to modulate the velocity of the bubble microrobot, which controls the orientation of the Janus microspheres and the direction of the jet flow by the bubbles. A series of studies have relied on chemical reactions in H_2_O_2_ solutions. However, this mechanism should be applicable to various solution systems if bubbles can be generated by other reactions. For example, it could be used in the human body for drug delivery in acidic environments such as the stomach.

**Figure 27:**
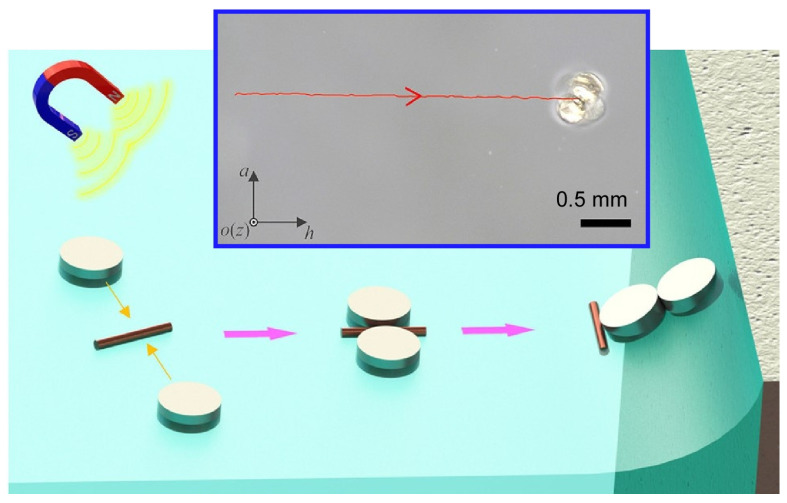
A pair of magnetic microdiscs as partners with flexible motion and in situ micromanipulation on an ethylene glycol surface controlled by adjusting the direction of the applied magnetic field. Reprinted with permission from Reference [206]. Copyright 2022 American Chemical Society.

Microrobots that operate on liquid surfaces have great potential for micromanipulation in confined spaces and confined locations. In particular, contraptions that produce agile and functional microrobots with simple structures are desired. Wang and co-workers reported a pair of magnetic microdiscs that work as partners (Figure 27) [206]. This pair of microdiscs allowed flexible motion and in situ micromanipulation on an ethylene glycol surface. The connection and separation of these microdiscs can be controlled by adjusting the direction of the applied magnetic field; the two microdiscs are connected and then converted into micro partners under an oscillating magnetic field. By varying the vertical component of the oscillating magnetic field, controllable propulsive forces such as paddling and wriggling could be achieved. The pair could also crawl on solid surfaces in liquids. As flexible microgrippers, they were demonstrated to operate on liquid surfaces, including capturing the target material, delivering it along a predetermined path, and releasing it.

Bryan et al. fabricated microswimmers by photolithography and electrodeposition using Co-Ni-P magnetic nanoparticles consisting of elastically and magnetically coupled hard Co-Ni-P and soft Co ferromagnets [207]. The self-propulsive behavior of the magnetoelastic composite microswimmers was verified at the air-water and water-substrate interfaces under a uniaxial magnetic field. The propulsion of the microswimmer is a combination of dipolar interactions between Co and Co-Ni-P magnets and rotational torque due to the applied magnetic field, plus elastic deformation and hydrodynamic interactions between different parts of the swimmer. Swimming speed and direction depend on the frequency and amplitude of the magnetic field. Yang and co-workers have demonstrated a self-powered microrobot inspired by a water strider that can harvest energy from water by means of superhydrophobic legs [208]. This water-strider-shaped microrobot can walk on the surface of water, light LEDs, and power temperature sensors without an external power source. Dynamic coupling of motors and shape memory alloys allows the microrobot to paddle its superhydrophobic legs on its side using collected energy. In addition, a power management circuit is integrated into the micro-robot, allowing it to light LEDs and supply power directly to the temperature sensor.

## 4. Perspectives

This review paper overviews several recent examples of research on molecular machines and microrobots, which are small objects that work in a variety of modalities. These, like machines in our macroscopic world, are important in how they architect their functional structures. Therefore, methodological approaches such as nanoarchitectonics and microfabrication will determine the performance of these tiny machines. This is the subject where nanoarchitectonics can show its true value.

These small machines, especially molecular machines, have been studied mainly for their work in solution and on solid surfaces. In this review paper, it is examined that the results obtained when the working field of these tiny machines is moved from these usual fields to the water surface. The behaviors of molecular machines are influenced significantly by the following characteristics of the air-water interface: (i) it is a phase boundary with different dielectric constants at the molecular level, (ii) the size spread is extremely different between the film lateral direction and the film thickness direction, (iii) it has a very high degree of freedom of motion compared to solid surfaces, (iv) collective behavior of aggregated molecules can occur, and so on. By placing molecular machines at the air-water interface, the scientific horizon and depth of molecular machine research will increase dramatically. In the field of microrobotics, more practical and advanced systems have been reported, such as the development of microrobots that act like swimming on the water surface (microswimmer), behave on the water surface like water striders and insects, and can transition between water, the water surface, and solid surfaces. These advanced examples of microrobots can be seen as examples of research that molecular machines will follow. It is expected that similar advanced systems that are close to practical use will be realized in molecular machines. The air-water interface and the thin films created at the interface can be used as simplified models of biological membranes and cell surfaces. The research currently being conducted on the surface of water may provide significant basic knowledge for practical use in cells. In fact, examples of molecular motors that work by acting on cell membranes have been reported.

Mankind is tackling the challenges it faces in energy [209,210,211], the environment [212,213,214], and biomedicine [215,216,217] problems through the use of a variety of materials and their nanostructures. While humans strive day and night, living organisms, the product of billions of years of evolution, perform functions far beyond that. Extremely superior functions, such as super-efficient energy production and extremely logical information transfer, are universally found in living systems [218,219]. This high functionality is due to the ultimate form of nanoarchitectonics, in which functional nanostructures are organized in a highly rational manner. More importantly, biological systems are very dynamic, with each functional unit moving and performing its function like a machine [220,221]. Therefore, as described in this paper, more advanced functional material systems are expected by applying the concept of nanoarchitectonics to molecular machines and microrobots. Ultimately, it is important to understand how to organize dynamic complex systems. Biological systems have evolved over a long period of time. However, humans have now developed high-speed information processing systems such as machine learning and materials informatics [222,223,224]. The use of these information technologies in materials science is flourishing. There have been proposed to integrate nanoarchitectonics and materials informatics [225,226]. One of the most desirable contributions of molecular machines and microrobots would be their practical uses in biomedical fields [227,228]. Because their uses in biological systems have huge working opportunities on soft interfaces. Therefore, investigation on molecular machines and microrobots at the air-water interface would give various fundamental knowledges for such biomedical uses. In addition, active surface coating with molecular machines and microrobots would be important key techniques. By using such tools, it is expected that the development of functional material systems by nanoarchitectonics, including molecular machines and microrobots, will make great progress.
